# Effect of Grain Refinement on the Corrosion Resistance of 316L Stainless Steel

**DOI:** 10.3390/ma14247517

**Published:** 2021-12-08

**Authors:** Ewa Ura-Bińczyk

**Affiliations:** Faculty of Materials Science and Technology, Warsaw University of Technology, ul. Wołoska 141, 02-747 Warsaw, Poland; ewa.ura@pw.edu.pl

**Keywords:** grain refinement, corrosion resistance, non-metallic inclusions, hydrostatic extrusion, 316L stainless steel

## Abstract

The effect of hydrostatic extrusion (HE) on the microstructure, uniform corrosion, and susceptibility to a localized attack of 316L stainless steel was studied. Both qualitative and quantitative analyses of inclusions before and after HE were carried out. The multiplication of non-metallic inclusions after HE lowered the stability of the passive film over a broad range of pH, while refinement of the matrix had a minor effect on it. The refined materials were prone to metastable pitting, but their pitting corrosion resistance was improved.

## 1. Introduction

Grain size refinement leads to an improvement in mechanical strength, as described by the Hall–Petch relation [1]. This can be achieved by severe plastic deformation (SPD), which brings about substantial microstructural changes, primarily grain size reduction and an increase in dislocation density, without changing the chemical composition [2,3,4]. Different SPD methods have already been successfully applied to the processing of stainless steel [5,6,7,8,9,10]. One of them is hydrostatic extrusion (HE), which gives rise to a uniform refinement of microstructure throughout the whole volume of relatively large samples [11]. Significant improvements in the mechanical strengths of 316L and 316LVM stainless steels following HE have previously been reported [8,9,10]. Although a great deal of attention has already been paid to the mechanical properties of stainless steels after bulk SPD, there are still few studies on the effect of structure refinement on their corrosion behavior.

In an environment that enables the formation of a passive film, microstructural refinement generally enhances the passivation ability of materials [12,13,14]. The defects in the structure (dislocations, grain boundaries, boundary junctions, shear bands, and deformation) act as active sites for passive film nucleation [13,15,16,17] and are multiplied in the refined structure. The higher density of defects accelerates passive film formation and changes its nucleation mechanism from progressive to instantaneous [13,18,19]. However, it also increases the number of defects in the passive film, which compromises its stability [19,20,21,22,23,24]. In fact, the improvement in passive behavior linked to higher Cr enrichment of refined materials is a result of a faster dissolution of Fe [13,25,26]. For warm-rolled 304 stainless steel [24], the deterioration in passive film stability was shown to be related to a high density of dislocations. After annealing, which reduced dislocation (but did not change the grain size), a significant improvement in the passivity of the refined 304 stainless steel was observed. Similar observations have been noted for materials after surface nanocrystallization [18,27,28] or bulk refinement [29], where, before annealing, the refinement process increased the passive current density while, following annealing, it improved the materials’ compactness and stability.

The pitting corrosion resistance of stainless steel is usually enhanced after grain refinement [26,30]. The refined structure promotes the occurrence of metastable pitting events while retarding stable pit growth due to its remarkable repassivation ability [31]. However, these findings were mostly observed with nanocrystalline stainless steel thin films [12,18,19,32,33,34], which were characterized by increased surface homogeneity and a lower number of inclusions (or their absence) [33,34]. Pan et al. [34] reported that Mn and S dissolved in a solid solution of α-Fe phase in a magnetron-sputtered thin film, and that pitting initiated at the grain boundaries. For bulk commercial 316L stainless steel, it is well recognized that manganese sulfide (MnS) and oxide inclusions trigger the onset of pitting corrosion [35,36,37,38]. During SPD, these inclusions are refined, but their size reduction is lower when compared to the refinement of the matrix. Results on the pitting corrosion resistance of bulk refined materials are often contradictory. A deterioration in pitting corrosion resistance was noted for 316L steel after dynamic plastic deformation (DPD) [29] and for 303 steel after HE [39]; it was linked to the fragmentation of non-metallic inclusions. However, 316L stainless steel, after HE, has shown improved resistance to localized attack [40]. Other studies have revealed that grain size had no effect on the breakdown potential of cold-rolled 304L steel [41].

So far, the influence of non-metallic inclusions on the corrosion behavior of austenitic steels after SPD has gained little attention. However, it seems that alteration in their size and distribution plays an important part in the corrosion resistance of SPD-refined materials. In our study, the uniform and localized corrosion of 316L stainless steel after HE is investigated, focusing on the effect of MnS and oxide inclusions in the refined structure.

## 2. Materials and Methods

### 2.1. Materials

The material studied was 316L austenitic stainless steel purchased from Sandvik Ltd. (Stockholm, Sweden). A nominal composition of 316L stainless steel is shown in Table 1. The chemical composition was measured using an X-ray fluorescence spectrometer (XRF) by Bruker S4 EXPLORER is listed in Table 2.

The material was delivered in the form of hot-extruded rods and was annealed to homogenize the microstructure.

**Figure 1:**
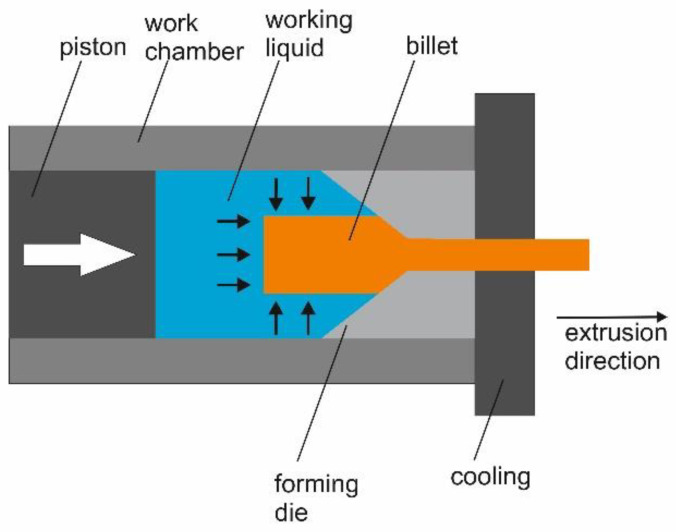
Scheme of the hydrostatic extrusion process.

Billets were subjected to an HE process, according to the procedure described in [42]. The 316L rods, with initial diameters of 8.9 and 11.4 mm (Table 3), were hydrostatically extruded in oil, in a multi-step process, to a final diameter of 6 mm. This process is characterized by high strain rates at a relatively low temperature. Plastic deformation was achieved accumulatively (step-by-step). A schematic diagram of the HE process is shown in Figure 1.

The accumulated strain was calculated using Equation (1):

(1) $$\varepsilon =2\times \ln\frac{d_{k}}{d_{p}}$$

where *d_p_* = initial diameter and *d_k_* = final diameter. The accumulated strain is listed in Table 3.

The sample, after annealing, was designated as coarse-grained (CG), and the extruded samples were designated as HE0.8 and HE1.3, where the numbers refer to the accumulated strain. Microscopic observations and electrochemical measurements were performed on cross-sections (cs) perpendicular to the HE direction and, on longitudinal sections (ls), parallel to the HE direction. The hydrostatic extrusion was carried out atthe Institute of High Pressure Physics, Polish Academy of Sciences, Warsaw, Poland. All the extrusion experimentswere conducted at ambient temperature.

### 2.2. Microstructural Observations

A Nikon Epiphot 200 light microscope (Nikon, Tokyo, Japan) was used to evaluate the microstructures of the investigated materials. For microscopic observations, the samples were polished to a mirror finish and etched with a solution of 50 mL 38% HCl, 25 mL 60% HNO_3_, and 25 mL H_2_O.

To characterize the inclusions formed in the material, a field emission scanning electron microscope (FE-SEM SU-70, Hitachi, Tokyo, Japan), equipped with an energy-dispersive X-ray spectrometer (EDS, Thermo Fisher Scientific, Waltham, MA, USA) was used. The inclusions were quantitatively described on both the cross and longitudinal sections in terms of their size and their number per area. The size of the inclusions observed on the cross-section was determined using an equivalent diameter, defined as the diameter of a circle with a surface area equal to the measured grain. In the case of inclusions on longitudinal sections, their minimum and maximum diameters were determined. At least 150 inclusions were measured in each case. The number of inclusions per unit area was calculated according to Equation (2):

(2) $$N_{A}=\frac{N}{A}$$

where *N*—number of inclusions and *A*—area.

### 2.3. Electrochemical Testing

Potentiodynamic polarization measurements were recorded using Autolab PGSTAT302N potentiostat/galvanostat (Metrohm, Herisau, Switzerland). A standard three-electrode setup, with a platinum sheet as the counter electrode, a saturated calomel electrode SCE as the reference electrode, and the sample as the working electrode, was used. Scans were recorded after 30 min of immersion, starting at the E_OCP_ with a 1 mV/s scan rate. Each measurement was repeated at least three times to ensure the reproducibility of the results. The measurements were carried out at an ambient temperature in unstirred test solutions.

Before each experiment, the surface of the samples was ground with 2500# SiC papers and then polished using polishing cloths with diamond suspensions (from 3 to 1 µm) until a mirror-bright surface was obtained. This surface preparation procedure was performed using an ATM Saphir 550 automatic polisher/grinder machine (ATM, Berlin, Germany). Finally, the surfaces were ultrasonically cleaned in ethanol.

Prior to each experiment, all solutions were freshly made from analytical grade reagents and distilled water. The chosen solutions covered a wide range of pH. Chloride ions were added to a buffer solution to evaluate the resistance to pitting corrosion, which ensured that a constant pH was maintained during measurements:

○ 0.5 M H_2_SO_4_ of pH 0.4
○ 0.11 M H_3_BO_3_ + 0.027M Na_2_B_4_O_7_ of pH 8.4
○ 0.5 M NaOH of pH 13
○ 0.11 M H_3_BO_3_ + 0.027M Na_2_B_4_O_7_ + 0.1 M NaCl of pH 8.4. Post-corrosion morphology observations were performed using a Nikon Epiphot 200 light microscope and TM1000 SEM (Hitachi, Tokyo, Japan).

## 3. Results

### 3.1. Grain Refinement

**Figure 2:**
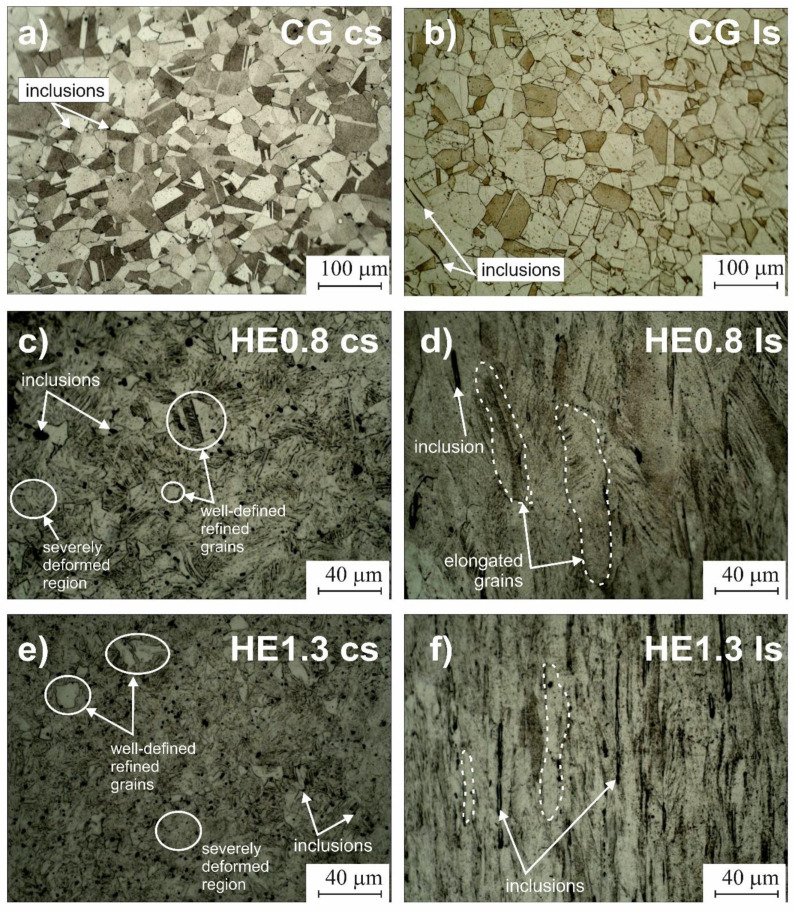
Light microscope observations of the microstructure of 316L stainless steel on the cross-section: (**a**) CG, (**c**) HE0.8 (**e**) HE1.3 samples and on the longitudinal section: (**b**) CG, (**d**) HE0.8 (**f**) HE1.3 samples.

The microstructures of the CG and HE samples are presented in Figure 2. Before processing, a coarse-grained microstructure with typical annealing twins was observed on the cross (Figure 2a) and longitudinal sections (Figure 2b). The apparent grain size, as well as the morphological texture, did not depend on the sample orientation. The measured average equivalent diameter of the grains was 30 µm. After HE, a significant microstructural refinement was observed. On the cross-sections, the heterogeneous microstructure with severely deformed regions, where no grain structure was visible, coexisted with well-defined grains of a markedly reduced size (Figure 2c,e). The microstructure on the longitudinal section (Figure 2d,f) consisted of elongated grains oriented parallel to the extrusion direction. Our previous investigations [8] revealed that these severely deformed regions to have a high density of defects, primarily deformation nano-twins of thickness between 50–200 nm and shear bands. Their volume fraction was estimated at 30% [8]. Due to the decay of primary grain boundaries, the microstructures of the HE0.8 and HE1.3 samples cannot be defined by means of equivalent grain diameter.

### 3.2. Inclusions

**Figure 3:**
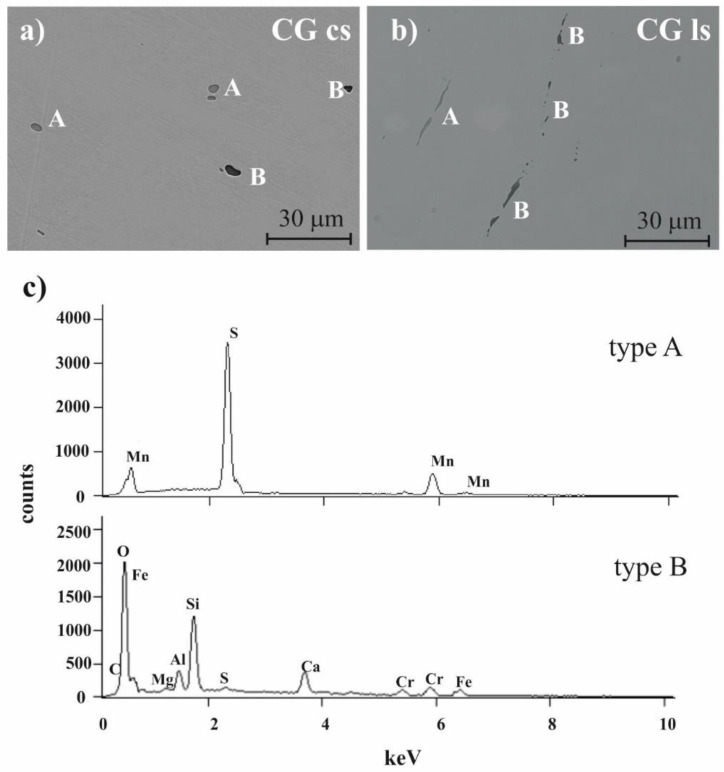
SEM observations of inclusions (**a**) on the cross and (**b**) longitudinal sections of CG sample and (**c**) EDS spectra examples of type A and B inclusions.

A characteristic feature of the microstructure of 316L stainless steel is the presence of intermetallic inclusions. Two types of inclusions were observed, as illustrated in Figure 3. Based on the chemical analysis of 20 inclusions (Table 4), the lighter (grey) inclusions and dark (black) inclusions were respectively identified as:

- MnS inclusions (designated as A in Figure 3)
- complex (Mg, Al, Si, Ca, Mn) oxides (designated as B in Figure 3).

For the CG sample, the shape of the inclusions was equiaxial on the cross-section (Figure 3a) and highly elongated on the longitudinal section (Figure 3b). Their appearance was a consequence of the production process and annealing having not changed their size or distribution. As the HE’s direction was parallel to the longitudinal section of the CG sample, the size of the inclusions was reduced on the cross-section, while, on the longitudinal section, they underwent simultaneous elongation and fragmentation.

The results of quantitative analysis of the number density (N_A_) and size of inclusions in the CG and HE samples on the cross-, and longitudinal sections are given in Table 5. The measurements were carried out for all inclusions, disregarding the differences in their chemical compositions. The average diameters of inclusions for the HE0.8 and HE1.3 cross-section samples were reduced by factors of 1.5 and 2.1, respectively, whereas their densities were 2.0- and 4.6-times higher. The inclusions on the longitudinal sections were less affected by HE. The overall number of inclusions per area increased by a factor of 1.3. The length of the inclusions was reduced slightly for HE0.8, while it was larger for HE1.3. Thus, the inclusions tended to be fragmented at the lower accumulated strain, while they were more likely to be elongated at the higher accumulated strain.

### 3.3. Uniform Corrosion

**Figure 4:**
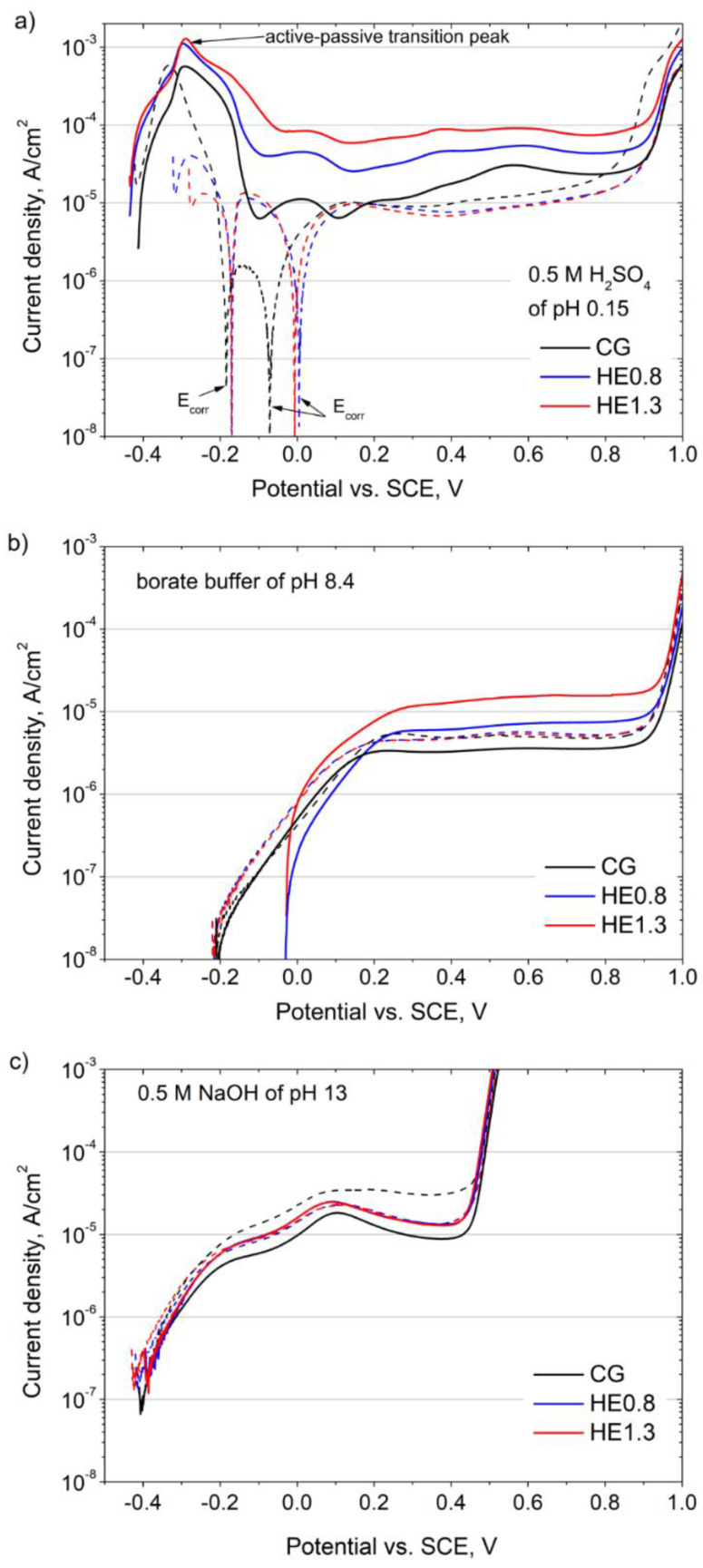
Anodic potentiodynamic polarization curves of the CG, HE0.8, and HE1.3 samples recorded in (**a**) 0.5 M H_2_SO_4_, (**b**) borate buffer (0.1 M H_3_BO_3_ + 0.024 M Na_2_B_4_O_7_) and (**c**) 0.5 M NaOH on cross-section (solid lines) and longitudinal section (dash lines).

The potentiodynamic curves recorded for the CG and HE samples are shown in Figure 4. The scans were recorded on the cross-section (solid lines) and longitudinal section (dashed lines).

In 0.5 M H_2_SO_4_, the general shape of the potentiodynamic curves differed for the cross-sections and longitudinal sections. On the cross-section of the CG sample, the observed active-passive transition peak was followed by a second anodic peak and the passive region (Figure 4a). A similar trend was observed for the HE samples; however, the anodic current density increased with the accumulated strain. The difference in the current density was most pronounced in the region between −0.1 and 0.8 V/Ref. For a potential of 0.1 V/Ref, the current density was 4.5 higher for the HE0.8 sample (27.4 µA/cm^2^) and 10 times higher for the HE1.3 sample (60.3 µA/cm^2^) when compared to the CG one (6.2 µA/cm^2^). On the longitudinal sections, the shape of the scan was different. Up to 0.1 V/Ref, significant changes in corrosion current were observed. The corrosion potential (*E_corr_*) was not stable during polarization, which indicates that the passive film was unstable. As the CG sample was annealed, the geometry of the inclusions was the only difference between the cross-section and longitudinal section. For the HE samples, the shape of the potentiodynamic curves was similar to that recorded for the CG one. The differences in the passive current density between the coarse material and the refined ones were less pronounced than was observed for the measurements performed on the cross-section.

In the borate buffer solution (Figure 4b), the shapes of the polarization curves recorded on the cross and the longitudinal sections were similar for all the tested samples: from *E_corr_* the current density increased up to the passive region. Significant differences in the anodic behavior were only observed on the cross sections of the examined materials: *E_corr_* of the refined materials was shifted to more positive values from −0.21 V/Ref to −0.03 V/Ref, and the current density (in the passive region at a potential of 0.30 V/Ref) increased from 3.5 µA/cm^2^ for the CG material to 6.6 µA/cm^2^ for HE0.8 and up to 13.7 µA/cm^2^ for HE1.3.

In the alkaline solution (0.5 M NaOH) (Figure 4c), the differences between the coarse-grained and refined materials were even smaller than in the borate buffer solution. The shape of the potentiodynamic polarization curves was the same for all the tested materials, and only the difference in the anodic current density was noticeable. It was the lowest for the cross-section of the CG sample, and it increased after HE. On the other hand, for the longitudinal sections, the anodic current density was highest for the CG sample and lower for the refined samples.

### 3.4. Localized Corrosion

**Figure 5:**
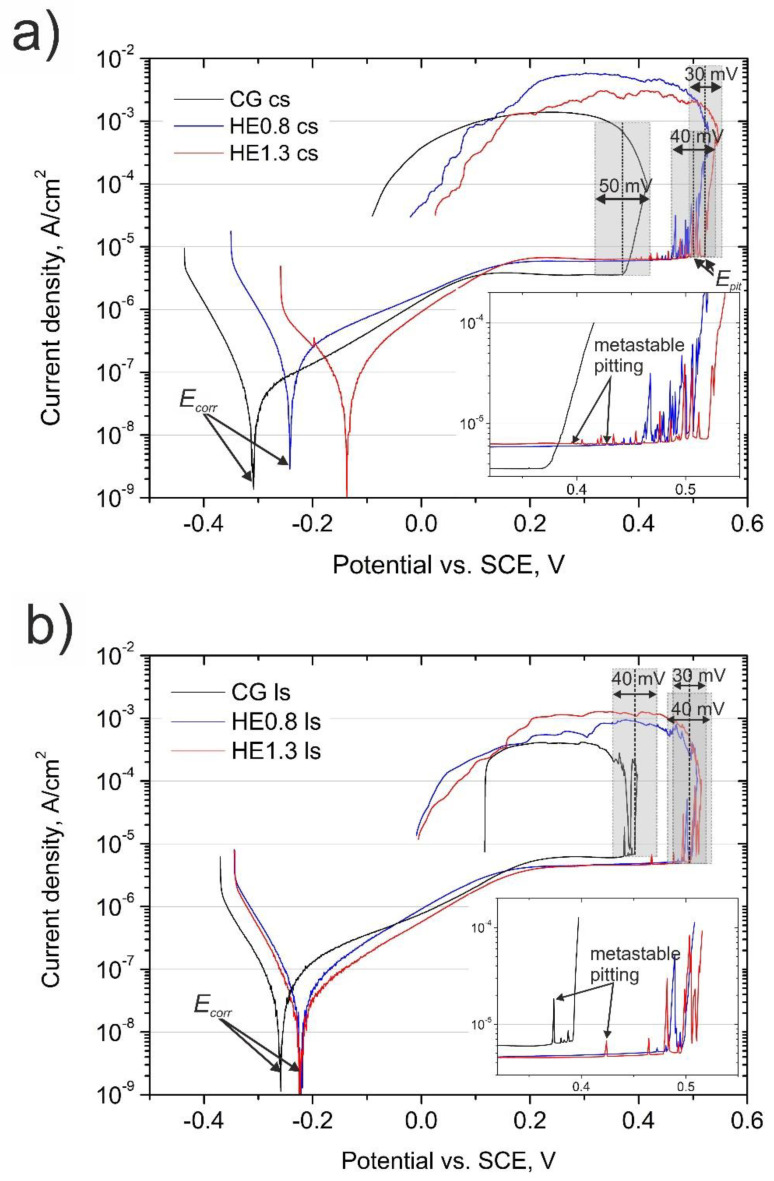
Anodic potentiodynamic polarization curves of CG, HE0.8 and HE1.3 samples recorded in borate buffer (0.1 M H_3_BO_3_ + 0.024 M Na_2_B_4_O_7_) with the addition of 0.1 M NaCl: (**a**) cross and (**b**) longitudinal section.

The susceptibility to localized attack was investigated in borate buffer solution containing chloride ions (0.1 M H_3_BO_3_ + 0.024 M Na_2_B_4_O_7_ + 0.1 M NaCl). The potentiodynamic curves recorded on the cross and longitudinal sections of CG and HE samples are shown in Figure 5.

The shape of all the curves is similar: a passive region followed by an abrupt current density increase related to pitting was revealed. On the cross-section, *E_corr_* shifted to more positive values, the passive current density (*i_pass_*) was higher, and the breakdown potential (*E_pit_*) also shifted to higher potentials, indicating an improvement in the corrosion resistance after HE. A significant difference between the CG and HE samples was the occurrence of metastable pitting on the refined samples. Although the pitting potential was highest for the HE1.3 sample (average value of 0.50 V/Ref), metastable pitting had already occurred at 0.39 V/Ref. On the longitudinal section of the refined materials, the shift of *E_corr_* and *E_pit_* to more positive values was also observed, while the *i_pass_* was slightly lower when compared to the CG sample. In addition, a larger number of anodic spikes before *E_pit_* indicated a higher frequency of metastable events on the refined samples.

**Figure 6:**
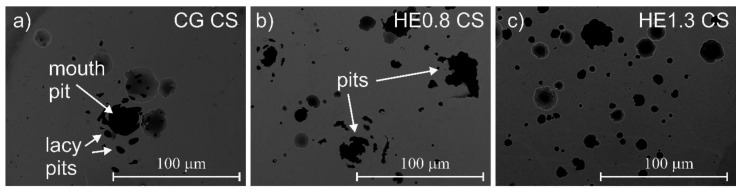
Post-corrosion morphology of the corrosion attack after potentiodynamic polarization in borate buffer solution with chloride ions on the cross-section of (**a**) CG, (**b**) HE0.8, and (**c**) HE1.3 samples.

**Figure 7:**
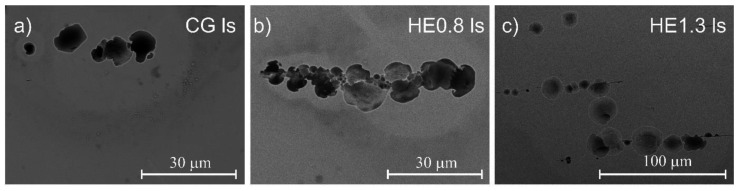
Post-corrosion morphology of the corrosion attack after potentiodynamic polarization in borate buffer solution with chloride ions on the longitudinal section of (**a**) CG, (**b**) HE0.8 and (**c**) HE1.3 samples.

The morphology of the localized attack after potentiodynamic polarization on the tested materials is shown in Figure 6 and Figure 7. The cross-section of the CG sample (Figure 6a) revealed the formation of a typical pit (mouth pit) with a number of small holes (lacy pits) distributed around it. Mouth pits with lacy pits on their peripheries were also observed on the HE0.8 sample (Figure 6b). For the HE1.3 sample, single round pits were observed. An important fact is the lack of lacy pits around the mouth pit; on the longitudinal section of the CG sample, the pits had a round shape (Figure 7a). After refinement, the pits were elongated in the extrusion direction (Figure 7b,c). Also, a higher number of larger pits was observed for the most refined sample (Figure 7c).

The number of pits per unit area for the tested materials is listed in Table 6. On the cross-section, the number of pits increased around three times after HE. On the longitudinal section, the number of pits was lower for the HE0.8 sample, while being higher for the HE1.3 sample. For the HE0.8 sample, this trend might relate to the fact that several pits around a single inclusion were connected, as shown in Figure 7b. For HE1.3, the pits were clearly formed along the extrusion direction, but they were not connected. It also seems that the pits on the longitudinal section were shallower after refinement.

## 4. Discussion

### 4.1. Effect of Grain Refinement on Passive Behavior

Previous microstructural studies on 316L stainless steel after HE revealed a severely deformed microstructure with a high density of defects, primarily dislocations, deformation twins of 50–200 nm thickness, and shear bands [8]. Additionally, new nano-sized grains were formed at the intersections of shear bands. On the longitudinal sections, the grains were elongated with some nano-twins within them, and their specific size ratio was greater than 20 [8]. It was proven that the multiplication of defects in the refined structure led to the growth of a more defective passive film [18,19]. The defective passive film had a higher ionic conductivity, which accelerated its growth but also decreased its stability and enhanced its dissolution (observed as an increase in passive current density). Recently, the lower stability of the passive film on warm-deformed 304 stainless steel [24] has been linked to its high dislocation density. After annealing at 950 °C, which reduced the volume fraction of dislocations, the passivity of the deformed samples was improved. The formation of the more defective passive film could be a possible explanation for the increase in passive current on the cross-sections of the HE0.8 and HE1.3 samples in acid solution. However, on the longitudinal section, the grain refinement had little or an opposite impact on passive film stability, as the observed anodic current was slightly lower after HE. In fact, the greatest difference in the passive film stability was observed between the cross and longitudinal sections of the CG sample. In these two sections of the CG sample, the microstructures, in terms of their grain size and texture, were similar, which is typical for annealed stainless steel. The only differences were the geometry and distribution of inclusions. The observed inclusions were equiaxial on the cross-section and elongated on the longitudinal section with respect to the extrusion direction. The elongated shape of the inclusions had an adverse effect on the stability of their passive films (multiple corrosion potentials). The number and morphology of the inclusions on the longitudinal section were preserved after HE. Therefore, the increase in the passive current density on the cross section after HE might be related, to some extent, to the multiplication of non-metallic inclusions. Additionally, the MnS inclusions dissolved at low pH, which may also have contributed to the total anodic current density [43]. In neutral and alkaline solutions, the differences between the CG and refined materials were smaller. Nonetheless, an increase in anodic currents was observed on the cross-section, while on the longitudinal section, a minor decrease in passive current was observed.

In our previous studies on the corrosion behavior of 316LVM after cold and hot HE, refinement increased the current in the active–passive transition peak, but no difference in current density was observed in the passive domain. In contrast to 316L steel, 316LVM is characterized by a very low number of non-metallic inclusions. Hara et al. [44] revealed that the passive current density for high-purity 316L with a reduced number of inclusions is one order of magnitude lower compared with the commercial one. Thus, the increase in the passive current density and the deterioration of the passive film stability on the cross-section after HE is related, to some extent, to the multiplication of non-metallic inclusions, while the refinement of the matrix has a minor effect on the overall anodic current density.

### 4.2. Effect of Grain Refinement on Susceptibility to Localized Attack

In aggressive solutions, the multiplication of inclusions after HE did not have an adverse effect on the resistance to localized attack, despite the fact that the number of pits was tripled. In fact, the corrosion potential was shifted to nobler values, and the breakdown potential was increased by 180 mV and 110 mV for the cross and longitudinal sections, respectively. For coarse-grained material, the breakdown potential for the cross and longitudinal sections of the CG sample were quite similar. Therefore, it can be assumed that the shape of the MnS inclusions did not have an effect on the pitting corrosion resistance. The important fact is that the pitting resistance was improved similarly on the cross and longitudinal sections after HE. Based on this, it might be assumed that the lower susceptibility of the HE materials to localized attack was related to the grain refinement.

The frequency of metastable pitting events was higher for the HE samples. This relationship indicates that grain refinement inhibited stable pit growth. This finding is in agreement with the literature and is explained by the higher repassivation ability of refined material [34]. Looking at the pits’ morphology on the cross-section of the CG sample, lacy pits were observed around the mouth pits’ peripheries. They could also be found on the HE0.8 sample, but only round-shaped pits were found on the HE1.3 sample. The lacy pits were the remnants of a lace cover formed during pitting [45]. The presence of a lacy, porous cover allows the stable growth of the pit, while the rupture of this cover leads to repassivation of the pit [46,47]. Isaacs and Kissel [48] found that pit propagation depended strongly on the state of the cover. Stressed cover with defects was easily ruptured, which led to pit repassivation, while thick cover protected pits until they were large enough to continue to grow without the cover. Perhaps the high density of defects after refinement led to the formation of a more defective and weaker lacy cover that could easily rupture. The less stable lacy cover easily collapsed, and the repassivation of the pit was more probable than its stable growth. Another interesting fact is that the pits had a similar round shape on both sections of the CG sample. However, on the longitudinal section of HE samples, the material had dissolved along the extrusion direction, and the pits were shallower. Since the pits initiated at the inclusions, it seems that the propagation of localized attack and the dissolution of the material occurred along with these inclusions. Most likely, the refinement of the matrix around the inclusions led to more lateral than a deep dissolution of the material.

## 5. Conclusions

The role of non-metallic inclusions in the electrochemical behavior of ultrafine-grained 316L stainless steel was investigated by potentiodynamic polarization and microstructural characterization. Based on the obtained results, the following conclusions can be drawn for ultrafine-grained 316L stainless steel:

(1) The stability of the passive film decreased with higher accumulated strain due to the multiplication of MnS and oxide inclusions over a wide range of pH; the refinement of the matrix has a minor effect on the passive film stability.

(2) The pitting potential was higher for the HE samples; therefore, the resistance to localized attack after HE is better compared to CG sample. At the same time, the frequency of metastable pitting events is higher for HE processed materials; however, the repassivation of metastable pits is more probable, and their stable growth starts at a higher potential.

## Figures and Tables

**Table 1:** Nominal chemical composition of 316L stainless steel.

Element	C	Si	Mn	P	S	Cr	Ni	Mo
% weight	≤0.030	≤0.75	≤2.00	≤0.040	≤0.030	16.5	11	2.1

**Table 2:** Chemical composition 316L stainless steel.

Element	Fe	Cr	Ni	Mo	Si	Co	Cu	V	C	Al	S	P
% weight	67.9	16.4	10.3	2.0	0.77	0.43	0.43	0.08	≤0.030	0.06	0.02	<0.4 ppm

**Table 3:** Cross-section reduction and accumulated strain.

Sample	Initial Diameter, mm	Final Diameter, mm	Accumulated Strain, ε
HE0.8	8.9	6.0	0.8
HE1.3	11.4	6.0	1.3

**Table 4:** Average chemical composition of inclusions types A and B.

Element, % Weight	O	Mg	Al	Si	S	Ca	Mn
Inclusion A	-	-	-	-	30.2–35.0	-	59.7–63.6
Inclusion B	32.5–53.0	0.5–2.4	1.9–7.5	10.6–29.7	0.9–2.3	8.1–39.2	16.0–27.1

**Table 5:** Quantitative analysis of inclusions on the cross-section and longitudinal section: N_A_—number of inclusions, d—average diameter, d_min_—average minimum diameter, d_max_—average maximum diameter.

Sample	N_A_, 1/mm^2^	d, µm	d_min_, µm	d_max_, µm
CG CS	1340	1.5 ± 1.2	-	-
HE0.8 CS	2620	1.0 ± 0.6	-	-
HE1.3 CS	6190	0.7 ± 0.4	-	-
CG LS	253	-	1.4 ± 1.1	8.0 ± 12.2
HE0.8 LS	331	-	1.0 ± 0.9	7.0 ± 12.2
HE1.3 LS	317	-	0.9 ± 0.5	10.3 ± 13.9

**Table 6:** Quantitative analysis of the number of pits per area on the cross-section (cs) and longitudinal section (ls).

Sample	NA, 1/mm^2^	
	cs	ls
CG	3.2	1.3
HE0.8	9.0	0.8
HE1.3	10.3	2.5

## Data Availability

Not applicable.
